# Effects of zinc supplementation on sexual behavior of male rats

**DOI:** 10.4103/0974-1208.57223

**Published:** 2009

**Authors:** DMAB Dissanayake, PS Wijesinghe, WD Ratnasooriya, S Wimalasena

**Affiliations:** Department of Obstetrics and Gynaecology, Faculty of Medicine, University of Kelaniya, Sri Lanka; 1 Department of Zoology, Faculty of Science, University of Colombo, Sri Lanka; 3 Department of Chemistry, Faculty of Science, University of Kelaniya, Sri Lanka

**Keywords:** Male rats, sexual competence, zinc

## Abstract

**CONTEXT:**

Effects of zinc on male sexual competence are poorly understood.

**AIM:**

To study the effects of different doses of zinc on the sexual competence of males using a rat model.

**MATERIALS AND METHODS:**

Three subsets (eight in each subset) of sexually experienced adult male rats were supplemented with three different oral doses of zinc sulphate (a daily dose of 1 mg, 5 mg and 10 mg respectively) for two weeks. A subset of eight animals without zinc supplementation was used as the control group Sexual behavior was observed by placing them individually in cages with receptive females.

**STATISTICAL ANALYSIS:**

Data analysis was done using SPSS v10 for windows computer software.

**RESULTS:**

Supplementation of 5 mg of zinc/day for two weeks led to a prolongation of ejaculatory latency; 711.6 sec. (SEM 85.47) *vs*. 489.50 sec. (SEM 67.66), *P* < 0.05 and an increase in number of penile thrusting; 52.80 (SEM 11.28) *vs*. 26.50 (SEM 6.17), *P* < 0.05, compared to controls. The same group had elevated prolactin (PRL) and testosterone (T) levels compared to controls at the end of treatment period; PRL- 7.22 ng/dl (SEM 3.68) vs. 2.90 ng/dl (SEM 0.34) and T- 8.21 ng/ml (SEM 6.09) *vs*. 2.39 ng/ml (SEM 1.79), *P* < 0.05. In contrast, reduction of libido was evident in the same group, but this effect was not statistically significant (*P* > 0.05). However, partner preference index was positive and 5 mg zinc supplementation did not exert a significant adverse effect on the muscle strength and co-ordination. The subset of rats supplemented with 1 mg/day did not show a difference from the control group while supplementation with 10 mg/day led to a reduction of the libido index, number of mounts and intromissions.

**CONCLUSIONS:**

Zinc therapy improves sexual competence of male rats; the effect is dose dependent. Increase in the T levels is beneficial in this regard. However, increase in PRL is responsible for the reduced libido index. Further studies on pigs and monkeys are needed to evaluate the therapeutic use of zinc in sexual dysfunction.

## INTRODUCTION

Zinc affects different aspects of mammalian reproduction. Testicular disruption, impaired spermatogenesis and subsequent poor semen parameters are found in males with zinc deficiency. Testicular concentration of zinc was lower in male sheep fed with zinc deficient diets. The same animals showed smaller seminiferous tubules and less lumen development than the controls.[1] Similarly variable degrees of maturation arrest in different stages of spermatogenesis with reduced diameter of seminiferous tubules were noted when rats were fed with zinc deficient diets.[2] Zinc deficiency causes a reduction in the structural parameters of seminiferous tubules influences serum levels of testosterone (T) and prolactin (PRL) in rats.[3,4]

On the other hand, high zinc levels have negative effects on sperm quality. Excessive zinc intake in mice have indicated a negative effect of increasing doses of zinc on sperm count and motility.[5] Although there have been studies focusing on various aspects of zinc related reproductive functions, studies on zinc related sexual behavioral aspects have received scant attention. In one study, intranasal irrigation with zinc sulphate has been reported to completely abolish the sexual behavior of male rats.[6]

The development of successful sexual behavior involves not only important neuroendocrine and local genital changes that begin at puberty, but also psychological and social influences that occur both before and after puberty.[7,8] Sexual behavior in males is regulated mainly by internal patterns of hormones; i.e. T, progesterone and PRL. These hormones are modulated by the male interactions with the social environment.[9]

In this study, the possibility of zinc as a component of some hormones, enzymes or regulating agents,[10] in influencing various aspects of sexual activity was evaluated by supplementation of rats with different doses of zinc sulphate (ZnSO_4_).

## MATERIALS AND METHODS

### Subjects

Wistar rats (From Medical Research Institute, Colombo) were obtained and kept in a well ventilated animal house under natural dark light cycle (temperature 28-30°C, humidity; 50-55%). Animals were housed in groups (four per group) until they reached sexual maturity (150-200 g). They were provided with pelleted food and water. Male rats were permitted access to receptive females on three separate occasions and then screened for sexual proficiency. Male rats who displayed consistently vigorous sexual activity were selected for the study.

### Zinc supplementation

Thirty two male rats were randomly assigned to four groups and submitted to one of the following daily treatment regimens: (a) 1 mg of ZnSO_4_ dissolved in 1 ml of distilled water. (b) 5 mg of ZnSO_4_ dissolved in 1 ml of distilled water (c) 10 mg of ZnSO_4_ dissolved in 1 ml of distilled water. Controls were provided with 1 ml of distilled water. Supplementation was done orally using a feeding tube. These daily regimens were continued for two weeks (daily at 17.00 hours) and housed two per cage.

### Observation of sexual behavior

Female rats were injected subcutaneously estradiol benzoate 12 μg in olive oil and 0.5 mg progesterone (Sigma chemicals, USA) in olive oil, 48 hrs and six hours prior to introduction to the males.[11] A cervical smear was observed under a light microscope and females in their estrous cycle were included in the study. Observations were performed during the dark phase of the day cycle (19.00 hours) under dim red light. After two hours of the last dose, rats were placed individually in transparent observation cages for 15 minutes adaptation period. A stimulus-receptive female was introduced to each male by gently dropping them in to the observation cage.

Measures of sexual behavior included: Mount and intromission frequencies and their latencies (the time from introduction of the stimulus female to first mount and intromission), ejaculatory latency (time interval between the intromission and ejaculation), penile thrusting number and index of libido (number of rats mated / number of rats paired).

The following parameters were computed using the observed behavioral measures. Copulatory efficiency (proportion of mounts resulting in vaginal penetration relative to the total number of mounts), intromission ratio (number of intromissions/number of intromissions + number of mounts) and intercopulatory intervals (average time between intromissions).[8]

Test was terminated after 15 minutes of observation or after ejaculation (whichever occurred first). Ejaculatory latencies were taken as 15 minutes when animals showed intromissions but failed to ejaculate during the test period. After analyzing the results, the following tests were performed with the group that indicated significantly positive effects compared to the controls:

#### Partner preference test

After two hours of zinc treatment, male rats were individually caged and kept for 15 minutes for adaptation. One estrous (receptive) and one diestrous (nonreceptive) female were introduced to each cage and the duration of physical contact with each female was recorded for 15 minutes. Partner preference index (PPI) was calculated as the difference between the time spent with estrous female and diestrous female. Positive indices indicate their positive sexual interest.[12]

#### Muscle strength and co-ordination

After two hours of treatment, males from each group (controls and 5 mg zinc treated) were tested for muscle strength using the bar test (time taken for the rat to fall from a hanging bar) and muscle co-ordination using the bridge test (time taken for the rat to slide off an angle bar when it is kept on the top of the bar).[12]

#### Testosterone and prolactin levels

Serum samples of the males of each group were assessed for T and PRL levels by the Elecsys 1010 analyzer using the method of electrochemiluminescence immunoassay. Inter and intra assay coefficient of variance for T was 3.2 % and 2.8 %, and for PRL 2.8 % and 3.2 % respectively.

### Statistics

The results are expressed as mean (SEM). Comparison between groups was made using Mann-Whitney U-test and Chi-Square test when appropriate. Data analysis was done using SPSS v10 for windows computer software. Statistical significance level was set at *P* < 0.05.

## RESULTS

### Effects of zinc on sexual behavior

A complete sexual behavioral cycle of males involved pre sexual performances, copulation and ultimate ejaculation. Pre sexual behavior comprises physical contact with the female, sniffing and licking of female genital area, licking of their own penis, and few mounts without intromissions. Although all induced females were screened for receptivity, some females showed a mild rejection in the beginning. However, there were no rejections when they were coupled with very active males. All animals which completed the behavioral cycle showed a normal pattern of behavior (not an aberrant sexual behaviour) during the observation period.

None of the parameters showed a significant difference between controls and the group treated with 1 mg of zinc. The percentage of males who engaged in intromission (% intromitted), was significantly reduced in 10 mg/day zinc group; only three animals showed the particular behavior. Similarly percentage of rats which ended up with ejaculation significantly decreased with the high dose (two out of eight). Libido index of the highest zinc treated group was significantly low compared to controls; (38 % *vs.* 88 %, *P* < 0.05). Number of mounts and intromissions was also significantly decreased in the same group; Number of mounts: 1.58 (SEM 3.16) *vs.* 11.0 (SEM 1.59) and number of intromissions 2.13 (SEM 4.27) *vs.* 11.0 (SEM 1.59), *P* < 0.05).

The number of animals ejaculating within 15 minutes was significantly reduced in 5 mg zinc treated group (one out of eight). However, all intromitted rats ejaculated between 20-30 minutes when observation was continued. Ejaculatory latency was significantly high in this group compared to controls; 711.60 Sec (SEM 85.47) *vs.* 489.50 Sec (SEM 67.66), *P* < 0.05. Similarly, they showed a significantly higher frequency of penile thrusting compared to controls; 26.50 (SEM 6.17) *vs.* 52.80 (SEM 11.28), *P* < 0.05 [Table 1].

**Table 1:** Effect of oral zinc supplementation on sexual behavior of male rats (n = 32)

Parameter	Control (n = 8)	1 mg/rat (n = 8)	5 mg/rat (n = 8)	10 mg/rat (n = 8)
Mounted %	100	100	100	100
Intromitted %	88	88	75	38*
Ejaculated %	100	75	25*	13*
Index of libido %	88	88	75	38*
Mounts, no. (SEM)	11.00 (1.59)	12.33 (3.84)	19.20 (4.36)	1.58 (3.16)*
Intromissions, no. (SEM)	10.50 (1.56)	11.60 (3.66)	18.60 (4.18)	2.13 (4.27)*
Penile thrusting, no. (SEM)	26.50 (6.17)	27.62 (7.17)	52.80 (11.28)*	21.53 (6.80)
Mount latency, sec. (SEM)	129.17 (29.56)	80.00 (20.00)	174.00 (27.50)	340.0 (189.20)
Intro.latency, sec. (SEM)	140.83 (29.79)	80.00 (20.00)	174.00 (27.50)	345.0 (187.14)
Ejac.latency, sec. (SEM)	489.5 (67.66)	640 (177.76)	711.6 (85.47)*	705.0 (141.50)
Copul. efficiency, % (SEM)	95.62 (3.28)	94.63 (3.21)	96.93 (1.46)	85.76 (8.10)
Inter copul. inter., sec. (SEM)	50.58 (7.78)	62.63 (34.93)	75.35 (21.23)	84.86 (12.60)

* P < 0.05, compared with controls Intro.latency – Intromission latency, Ejac. Latency – Ejaculatory latency, Copul efficiency – Copulatory efficiency, Inter. copul. Inter. – Inter copulatory intervals, Sec. – Seconds

There was a mild decrease in percentage of intromission and libido index in the 5 mg/day group, but it was not statistically significant.

### Effects of zinc on partner preference test

Partner preference index in both 5 mg zinc treated group and controls was positive. In the control group, time spent by males, with estrous and diestrous females, was 21.75 sec. (SEM 2.26) and 13.62 sec. (SEM 1.05) respectively. In the zinc treated group they spent 20.87 sec. (2.09 SEM) with estrous and 14.37 sec. (SEM 0.70) with diestrous females. The PPI of controls and zinc treated group was 8.12 Sec. (SEM 2.32) and 6.50 Sec. (SEM 1.76) respectively. There was no significant difference between the two groups, *P* > 0.05.

### Effects of zinc on muscle strength and co-ordination

Zinc supplementation (5 mg/day) did not exert a significant adverse effect on the muscle strength assessed by the bar test (control *vs.* treatment; 43.13 Sec (SEM 13.03) and 43 Sec (SEM 15.65), *P* > 0.05, and muscle coordination assessed by the bridge test (control *vs.* treatment; 62.60 Sec (SEM 39.79) and 55 Sec (SEM 8.06), *P* > 0.05.

### Effects of zinc on serum prolactin and testosterone levels

Both serum PRL and T levels were comparable between controls and zinc treated groups (5 mg/day), assessed after three hours of treatment in the first day. Zinc treatment for two weeks caused a significant (*P* < 0.05) increase in both serum levels of T and PRL [Table 2] compared to the control group.

**Table 2:** Serum levels of prolactin and testosterone in male rats before and after treatment with zinc sulphate 5 mg/day

Treatment	PRL ng/dL (SEM) (n = 8)	T ng/mL (SEM) (n = 8)
Control (day 1)	2.29 (1.27)	1.84 (0.92)
Treated (day 1)	2.90 (0.34)	2.39 (1.79)
Control (day 14)	2.20 (0.64)	1.57 (0.53)
Treated (day 14)	7.22 (3.68)*	8.21 (6.09)*

* P < 0.05, compared to control (day 1)

## DISCUSSION

We studied the involvement of zinc in the sexual behavioral response of male rats. The study design employed a rat model to predict the human sexual response to elemental zinc supplementation. Rats were used because they are very social and copulate under a variety of circumstances, regardless of the presence of a human experimenter. They are practical (small and easy to handle) and certain tissues and neuroendocrine systems are strikingly similar to humans.[13]

Parameters evaluated in this study are determinants of some important aspects of the male sexual competence. Libido index, intromission and mount latencies are indicators of libido, arousability and motivation. Copulatory efficiency and inter-copulatory intervals are indicators of sexual vigor, while penile thrusting is an indicator of penile erection.[8,11,14,15] Our results showed that the libido index was significantly reduced in the 10 mg/day zinc saulphate treated group. In the same group, majority of the animals (62%) failed to complete their sexual behavioral cycle within the observed period. Though these unsuccessful rats showed the initial steps of the cycle such as licking, physical contacts with the females and few mounts and intromissions, they were inactive at later stages. Therefore a significant reduction of the libido index, number of intromissions, mounts, and ejaculations were observed in the high dose of zinc treated group compared to controls.

The group treated with the lower concentration of zinc (1 mg/day) did not show an alteration in any of the observed parameters. However, supplementation with a dose of 5 mg/day per rat caused substantial prolonged ejaculatory latency and increased in number of penile thrusting. The other parameters studied remained unchanged indicating uninterrupted libido, sex vigor and performance. Majority of male rats (75 %) showed the prominent actions of sexual behaviour (mount, intromission and penile thrusting) and did not ejaculate within the 15-minute observation period.

Though higher doses of zinc reduce libido, supplementation with a medium dose (5 mg/day) has some beneficial effect on the sexual competence of adult male rats. The major significant effects of this dose of zinc are prolongation of ejaculatory latency without disturbing sexual arousability, motivation, penile erection and sex vigor. Also, the partner preference index of the 5 mg/day group was positive and comparable to the controls. A positive partner preference index is indicative of unchanged sexual interest of males.[16] These results confirmed that libido and sexual interest are not affected by zinc supplementation with a 5 mg/day dose. However, mild reduction in percentage of intromission was observed in this group and it is postulated that this may be situational rather than an effect of supplemented zinc. This is based on our observation where mild rejection by the females at the initial phase of the behavior led some males to refrain from sexual activity.

Reduction of the libido index was the major disadvantage that we observed with zinc supplementation. Substances that affect libido usually act centrally and may reduce desire by causing sedation or hormonal disturbances.[17] The role of elevated levels of PRL in serum as an inhibitor of sexual drive and gonadal function is well established.[18] This reduction of sex drive may occur through the modification of activity of dopaminergic neurons in the CNS that are regarded as controlling sexual motivation and function.[19] Our study demonstrated a significant increase of serum PRL level (2.9 to 7.22 ng/dl) within two weeks of supplementation of zinc (5 mg/day). This is a possible explanation for the reduced libido with increasing doses of zinc observed in this study.

In a study with human subjects, Kruger *et al*. have reported that acute changes in the normal physiological levels of PRL led to a significantly prolonged ejaculatory latency, but minor reductions of sexual drive and function.[19] Although zinc induced elevation of PRL was not an acute effect, findings similar to the human study (prolongation of ejaculation and mild reduction of libido index with medium dose of zinc) were observed in this study. However, the prolongation of ejaculatory latency may not be merely due to effects of elevated PRL because elevated PRL levels are known to be associated with the negative aspects of sexual activities (decreased sexual desire and frequency of sexual intercourse).[20]

Although we observed an elevation of serum PRL levels with supplementation of zinc, in most studies on humans, the relationship between these two parameters is inverse.[21,22] However, there are no published data on rats to support our finding.

In male rats, main olfactory epithelium (MOE) exerts an important role in regulating sexual behavior. Intranasal irrigation with zinc sulphate has been reported to destroy the MOE and completely abolish the sex behavior.[6] In this study supplementation of zinc was done using a feeding tube and precautions were taken to avoid contacting nasal area. Hence the possibility of reducing sexual performance due to MOE disturbance is ruled out. Some humans experience gastrointestinal irritation with supplementation of zinc.[23] If the same is applicable to animals it may be another possible explanation for the reduction of libido index with elevated doses of zinc. One drawback of our study is that we did not compare the weight of animals before and after treatment.

The prolongation of ejaculatory latency may be beneficial when present with unimpaired arousability, penile erection and sex vigor. The ejaculatory latency can also be prolonged due to some disorders in the neuroendocrine or reproductive system.[24] But the duration of zinc supplementation in our study was only two weeks which is not long enough to have an impact on the neuroendocrine or reproductive system.

The number of intromissions in the 5 mg/day group was comparable with controls. On the other hand number of penile thrusting was significantly increased with the same dose. These are indications of unimpaired erectile function due to supplemented zinc. Our observations also confirmed the lack of aberrant sexual behavior which is indicative of uninhibited penile tactile sensations.[15] Sexual behavior may also be changed with motor weaknesses and incoordination.[12] Supplementation with zinc did not impair muscle strength or coordination when tested with bar and bridge tests, indicating that prolongation of ejaculation was not mediated through these mechanisms.

In the present study zinc caused an elevation of T. This showed an increase from 2.39 to 8.21 ng/dl after two weeks of zinc treatment. This elevated T level may have contributed to the increase in number of penile thrusting (from 26.5 to 52.8) observed. Supplementation with 459 μmol/day of zinc for three months, in marginally zinc deficient healthy elderly men, has been shown to increase the levels of serum T from 8.3 to 16 ng/dl.[25] Laboratory experiments indicate that the nitric oxide erectile pathway is T dependent.[26] Many studies using animal models have confirmed that T is important in modulating the central and peripheral regulation of erectile dysfunction. T deprivation has a negative impact on the structure of penile tissues and erectile nerves.[27] Thus, elevated T levels subsequent to zinc supplementation may increase the sexual competence via rigid and sustained erection. This may promote greater tactile stimulation of the penis due to increased contact with vagina.[25]

## CONCLUSION

Zinc therapy (5 mg/day) improves sexual competence by increasing penile thrusting and prolonging ejaculatory latency without disturbing arousability and motivation of male rats. Increase in the T levels observed with zinc supplementation is beneficial in this regard. However, increase in PRL is responsible for the reduced libido index. Further studies on pigs and monkeys are needed to evaluate the possible therapeutic use of zinc in sexual dysfunction.
